# Are dental magnetic resonance imaging and ultrasonography techniques reliable alternatives for treatment planning dental implants? A systematic review and meta-analysis

**DOI:** 10.1186/s40729-025-00634-6

**Published:** 2025-08-11

**Authors:** Hengjia Zhang, Joe Donaldson, Vitor C. M. Neves, James Scott

**Affiliations:** 1 Department of Restorative Dentistry, School of Clinical Dentistry, University of Sheffield, Sheffield, UK; 2 Charles Clifford Dental Hospital, Sheffield Teaching Hospitals, NHS Foundation Trust, Sheffield, UK

**Keywords:** Dental implants, MRI, Ultrasonography, Systematic review, Implant planning accuracy, Cone-Beam Computed Tomography (CBCT)

## Abstract

**Background:**

The rising global demand for dental implant highlights the necessity for precise imaging techniques that minimise patient risk of radiation exposure. While the cone -beam Computed Tomography (CBCT) remains the gold standard, its ionizing radiation exposure raises safety concerns. This systematic review and meta-analysis aim to evaluate the accuracy of non-ionizing alternatives, Magnetic Resonance Imaging (MRI) and Ultrasonography (US), in dental implantology.

**Methods:**

Databases (MEDLINE, Scopus, Cochrane) were searched for studies (2014–2024) using predefined PICO criteria. Risk of bias was assessed via QUADAS-2. Meta-analysis employed fixed/random-effects models to synthesize quantitative data on geometric deviations and soft-tissue accuracy.

**Results:**

Twelve studies were included in this study. While MRI generally exhibited greater deviation in implant tip placement at 0.3 mm (95% CI -0.08, 0.68), its overall accuracy remained comparable to CBCT. MRI showed a higher mean deviation at the implant entry level of 0.38 mm (95% CI 0.04, 0.71) and for implant angulation with a mean difference of 0.81 degree (95% CI -0.50, 2.12), indicating less precision under specific conditions. Conversely, Ultrasonography demonstrated superior performance in soft tissue accuracy with a smaller deviation compared to CBCT, at just 0.04 mm (95% CI -0.04, 0.13).

**Conclusion:**

MRI and ultrasonography offer reliable non-ionizing alternatives for dental implant planning, with MRI matching CBCT in hard-tissue accuracy and ultrasonography excelling in soft tissue assessment. Further standardisation of protocols is needed to address variability in clinical workflows.

**Clinical trial number:**

The Clinical Trial Number is not applicable in this systematic review. This study was prospectively registered on the International Prospective Register of Systematic Reviews (PROSPERO) online with the identification number CRD42024610741.

**Supplementary Information:**

The online version contains supplementary material available at 10.1186/s40729-025-00634-6.

## Introduction

Dental implants, through osseointegration with the alveolar bone, have become the gold standard for restoration of missing teeth in aging population [1]. More than 9 million implants were placed globally in 2021, reflecting a market value exceeding $4 billion [2]. However, implant success relies heavily on precise preoperative imaging to avoid complications such as nerve damage (occurring in 1–6% of cases) or sinus perforation [3]. Cone-beam Computed Tomography (CBCT) is utilised for 3-dimensional bone assessment but repeated ionising radiation exposure can heighten the risk of radiation-induced health complications [4–6]. While single scans pose minimal risk, cumulative exposure from repeated procedures raises concerns about the significant risk of stochastic cancer, particularly when exceeding 100 mSv [7–9]. As such, non-ionizing imaging options, including Magnetic Resonance Imaging (MRI), Ultrasonography, optical scanning, spectrophotometry, and Optical Coherence Tomography (OCT), could be considered preferential for patient health.

MRI offers significant potential for assessing soft tissue and bone defect morphologies without radiation, providing comparable accuracy to CBCT anatomical structures and pathological processes in the maxillofacial area [9–11]. Additionally, ultrasonography provides real-time soft tissue measurement with submillimetre precision, which is critical for assessing mucosal thickness and peri-implant health [12, 13]. Despite these advances, no systematic review has comprehensively evaluated non-ionising imaging for implant planning. This study addresses this gap by analysing the diagnostic accuracy of MRI and ultrasonography compared to CBCT across preoperative and postoperative applications.

## Materials and methods

### Protocol and registration

The methodological approach of this review is aligned with the Preferred Reporting Items for Systematic Reviews and Meta-Analyses (PRISMA) guidelines. This study was prospectively registered on the International Prospective Register of Systematic Reviews (PROSPERO) online with the identification number CRD42024610741.

### Aim

This review addresses the focused question: Do non-ionizing dental imaging techniques provide adequate diagnostic information for pre- and post-dental implant surgery? A systematic and comprehensive search was conducted to identify relevant studies structured around the following PICO:

- **Population**: Human subjects and in-vivo models, including healthy volunteers and patients undergoing perioperative dental imaging for pre-implant planning and evaluation of post-implant pathologies.
- **Intervention**: Non-ionizing dental imaging techniques, specifically Magnetic Resonance Imaging (MRI) and Ultrasonography, with or without the enhancement of artificial intelligence.
- **Comparison**: Conventional radiological methods (Orthopantomography, Cone Beam Computed Tomography, or Computed Tomography), where available.
- **Outcome**: Evaluation of radiographic assessments’ feasibility and diagnostic accuracy in dental implant surgery.

### Study selection and eligibility criteria

Once the aims and objectives of the study were established, the University of Sheffield Library Service (A.T.) was enlisted on August 20, 2024, to assist in developing the search strategy. This strategy incorporated Boolean operators and MeSH (Medical Subject Headings) terms, optimized for databases including MEDLINE, Biosis, Scopus, and the Cochrane Library (Central). The optimized MeSH terms tailored to the PICOs are detailed in Table 1. All results from the database searches have been included in Supplementary Material 1 for reference.

**Table 1:** Search strategies corresponding to PICOs

Search Strategies	
Population	#1— “dental implants” OR “dental implant” OR “dental implantology” OR “titanium implant” OR “peri-implant” OR bone augmentation OR bone graft OR bone reconstruction OR sinus lift OR sinus lifting OR permanent dental restoration (inferior alveolar nerve [MeSH]) OR (lingual nerve [MeSH]) OR (mandibular nerve [MeSH]) OR (trigeminal nerve [MeSH]))
Intervention	#2— ((magnetic resonance imaging [MeSH]) OR (MRI) OR (nuclear magnetic resonance imaging [MeSH]) OR (NMR) OR (diffusion tensor imaging [MeSH]) OR (maxillofacial imaging) OR (ultrasonography)) #3— ((visualization) OR (neurography)) #4---- ((algorithm) OR (CNN) OR (convolutional neural network) OR (convolutional) OR (neural network) OR (Artificial intelligence) OR (artifact reduction) OR (image processing))
Comparison	
Outcome	#5— ((accuracy) OR (feasibility) OR (signal-to-noise-ratio [MeSH]))
Search Combination (s)	(#1) AND (#2 or #3 or #5) AND (#4)

Studies included in this review were selected based on the following inclusion criteria (Fig. 1):

1. Studies published in English.
2. Original research articles.
3. Human or in-vivo studies involving healthy participants or patients undergoing non-ionizing imaging for either mandibular or maxillary dental implant surgery. This includes pre-surgical planning and post-operative evaluation of pathologies.
4. Publications from the past 10 years.
5. Studies with full-text availability.

The exclusion criteria were as follows:

1. Studies lacking accessible full-text versions.
2. Review articles, letters to the editor, and case reports or case series with fewer than eight cases (excluding pilot studies without specific exclusion requirements).
3. Studies focusing solely on metal artefact reduction techniques.
4. Studies not clinically relevant to dental implant surgery.

All data were imported into EndNote™ 21 (Charles Clifford Dental Hospital 3162158872, EndNote 21.4 Build 20467), after which duplicate records were removed. Two reviewers (H.Z. and J.D.) independently performed the initial screening. The initial screening was conducted on all titles and abstracts to calibrate inter-reviewer reliability. Any disagreements were resolved through discussion, with the final author (J.S.) chairing the resolution process. Disagreement was again discussed and chaired by the third author (J.S.). The search strategy for all databases is reported in Supplementary material.

### Data extraction and collection

Two reviewers (H.Z. and J.D.) extracted data into a standardized excel template using a standardised form in Microsoft Excel 2024 for Mac (Version 16.90 Licence 2410387 Microsoft 365 subscription) with outcomes reviewed by J.S. The following data were composited on a shared spreadsheet in Excel format, with all the included studies containing the following: author, year of publication, country, study type, mean age, study objectives, imaging techniques, device used, company providing the machine, number and type of implant, outcomes, number of patients, and diagnostic accuracy.

### Quality assessment of studies

Two review authors (H.Z. and J.D.) performed the quality assessment by the Quality Assessment of Diagnostic Accuracy Studies (QUADAS-2) [14]. The two reviewers (H.Z. and J.D.) conducted their quality assessment independently, and the detailed spreadsheet and summary results were shared to each other. Discrepancies were discussed and resolved with the third reviewer (J.S.). In the evaluation of the included studies, four domains were considered: patient selection, index test, reference standard, and flow and timing, which were detailed in Fig. 2a and b. The risk of bias was categorized as follows:

- **Low risk of bias**: All criteria were met, presenting no significant potential for bias that could seriously affect the outcomes.
- **Some concerns**: Some aspects were not clearly articulated, potentially causing manageable effects on the reliability of the results.
- **High risk of bias**: Substantial issues were present that could drastically impact the study’s findings.
- **No information**: Certain details were omitted in the documentation of the studies; however, these omissions are not expected to have severe effects on the results.

### Statistical analysis

The quantitative analysis was performed using RevMan 5.4. Heterogeneity among the studies was assessed using the I² statistic and Cochran’s Q test. Heterogeneity was considered low if I² values were less than 25%, moderate if values were greater than 25% but less than 50%, and high if values exceeded 50%. Results were presented visibly in forest plots, with corresponding confidence intervals and a statistical significance established at *p* < 0.05.

### Descriptive analysis

Where heterogeneity prevents statistical analysis, descriptive statistical and narrative analysis was employed.

## Results

**Fig. 1:**
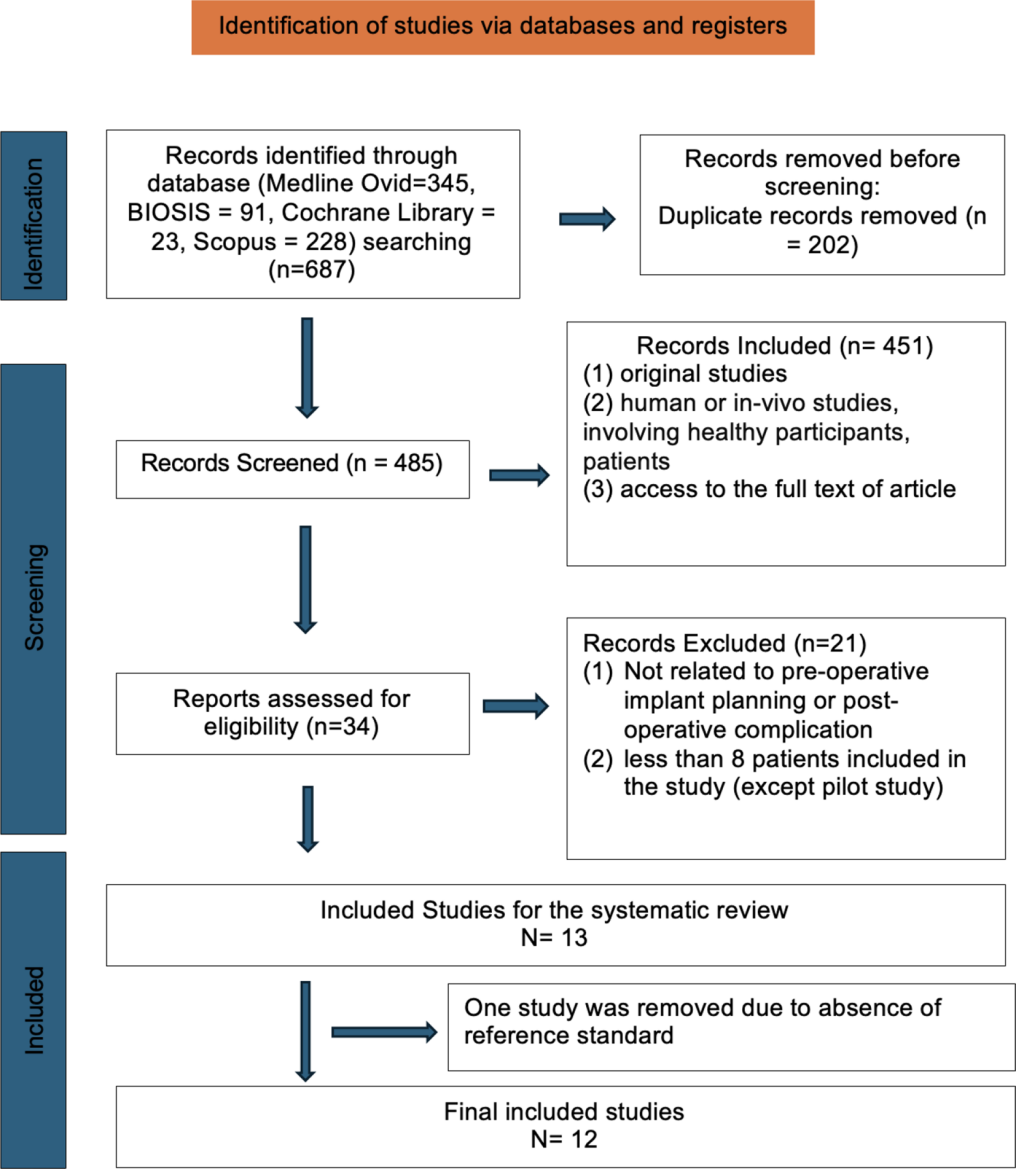
PRISMA flow diagram showing the article selection in this systematic review

### Study characteristics

From an initial pool of 687 potentially relevant articles, 12 studies met the inclusion criteria (Table 2). Three studies consistently applied MRI and CBCT to assess reproducibility and geometric reproducibility, with most referencing actual surgical outcomes as the standard [15–17]. Seven studies focused on evaluating the efficacy of various imaging techniques—including MRI, CBCT, and Ultrasonography(US)—in dental procedures, emphasising accuracy, reliability, and clinical utility to provide relative clinical information such as the location of inferior alveolar nerve, soft tissue thickness, and to improve the accuracy of information collection [18–24].

**Table 2:**
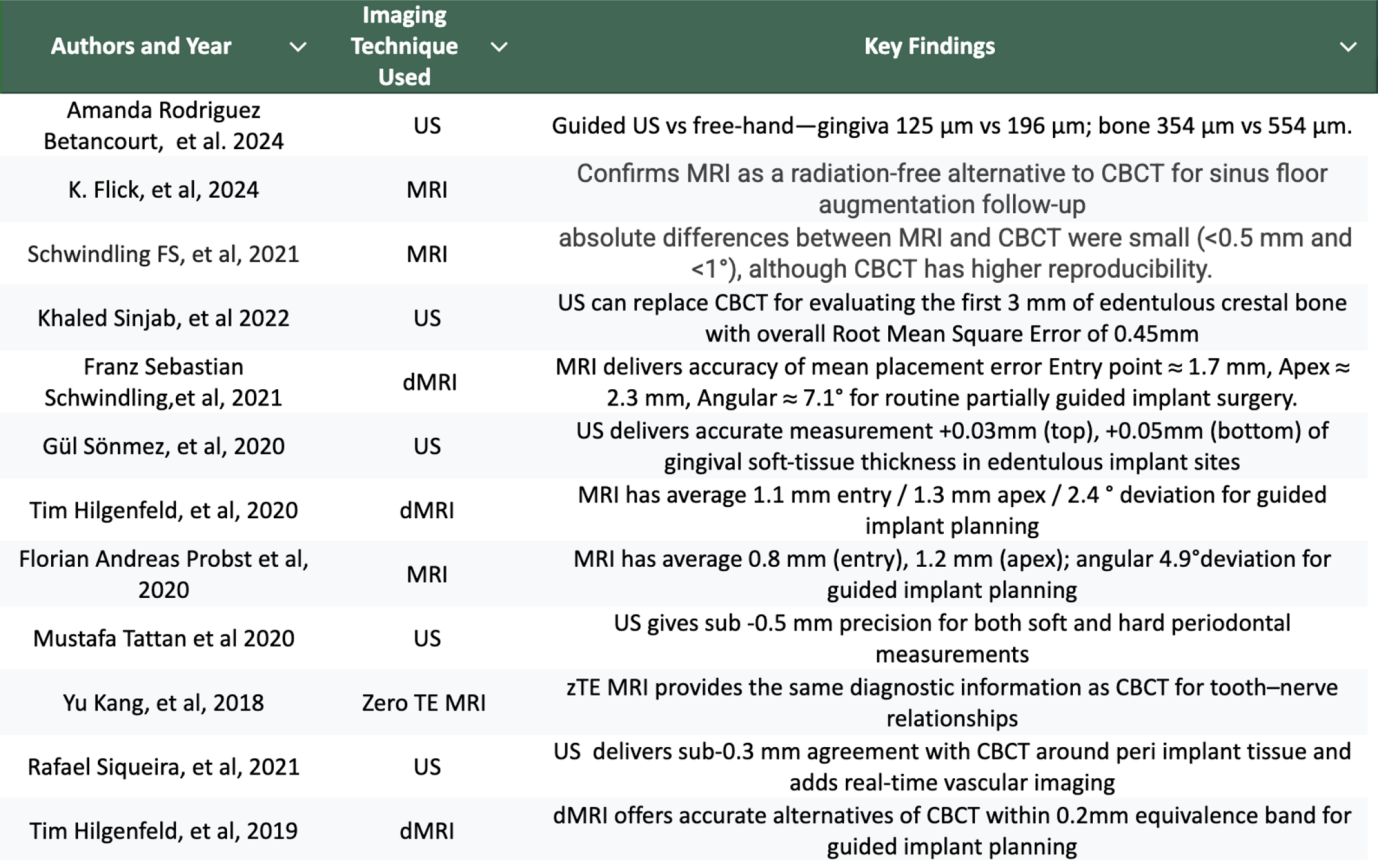
Characteristics of included studies

### Risk-of-bias assessment and quality assessment of studies

**Fig. 2:**
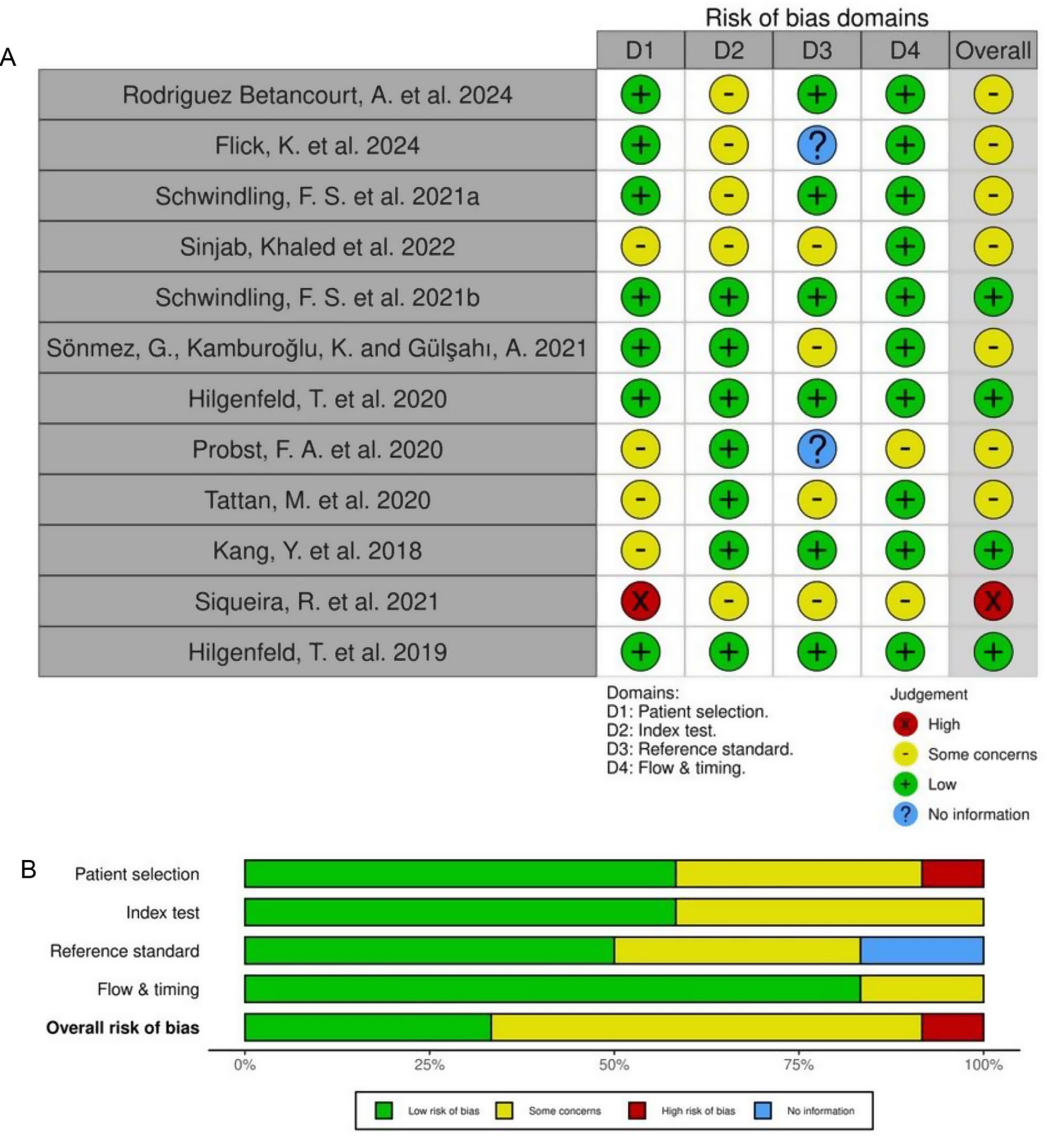
**A**: QUADAS-2 Risk of Bias Assessment with “traffic-light” plot display. **B**: Stacked bar chart summarising the proportion of studies classified as low risk, some concerns, high risk

The QUADAS-2 evaluation (Fig. 2a) includes one study with a high risk of bias, seven with some concerns, and four with a low risk. Figure 2b summarises overall quality. The main validity concern included insufficient reporting of patient selection methods- approximately 50% of the studies failed to clarify randomisation protocols, introducing potential sampling bias. One study exclusively enrolled patients with multiple implants, significantly increasing the potential for sampling bias. Additionally, 60% of studies lacked clear timelines for performing index tests and reference measurements, raising observation bias due to unblinded outcome interpretation.

### Synthesis of results for meta-analysis

**Fig. 3:**
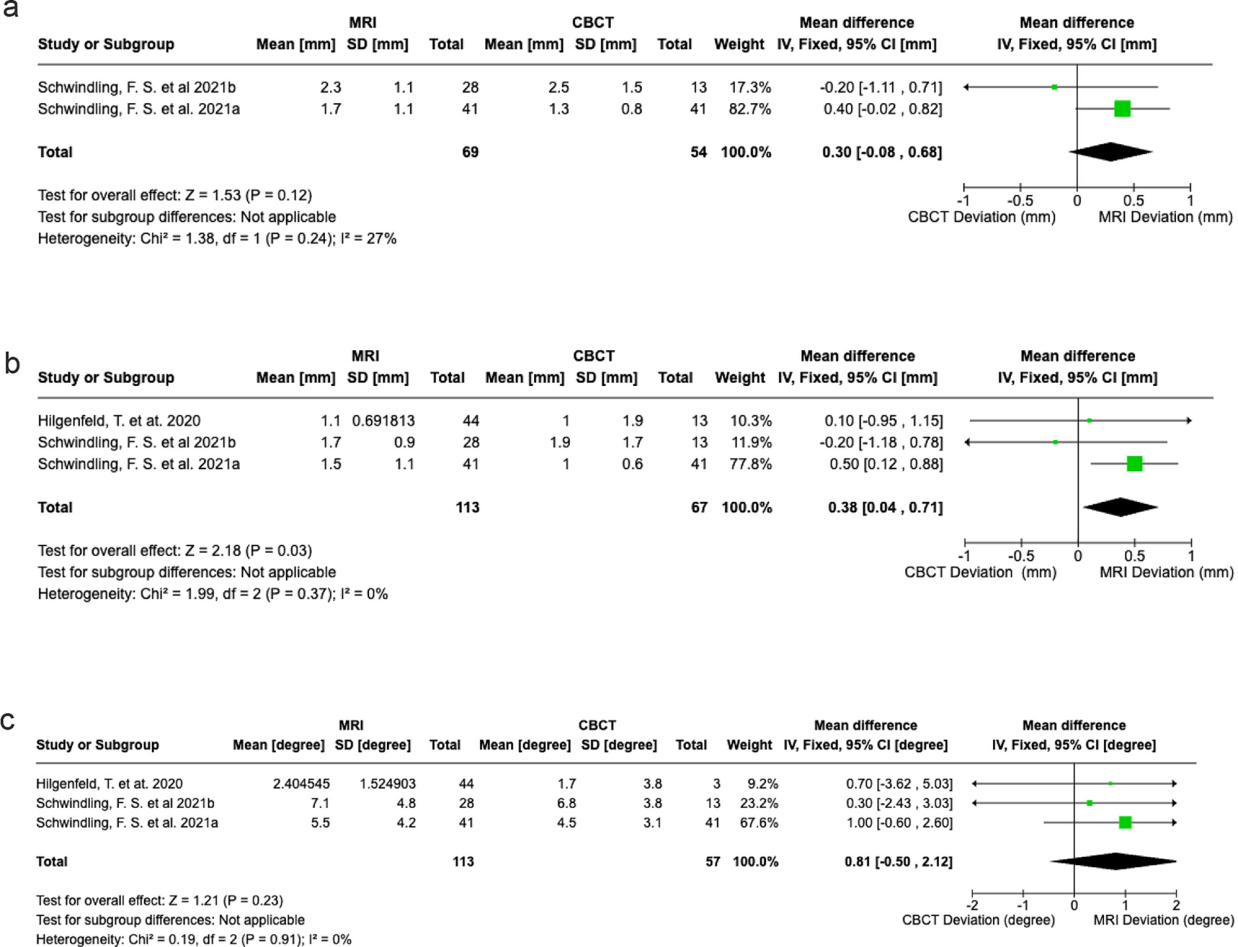
**a**: The mean deviation of implant tip. **b**: The mean deviation of entry level. **c**: The mean deviation of implant angulation

**Fig. 4:**
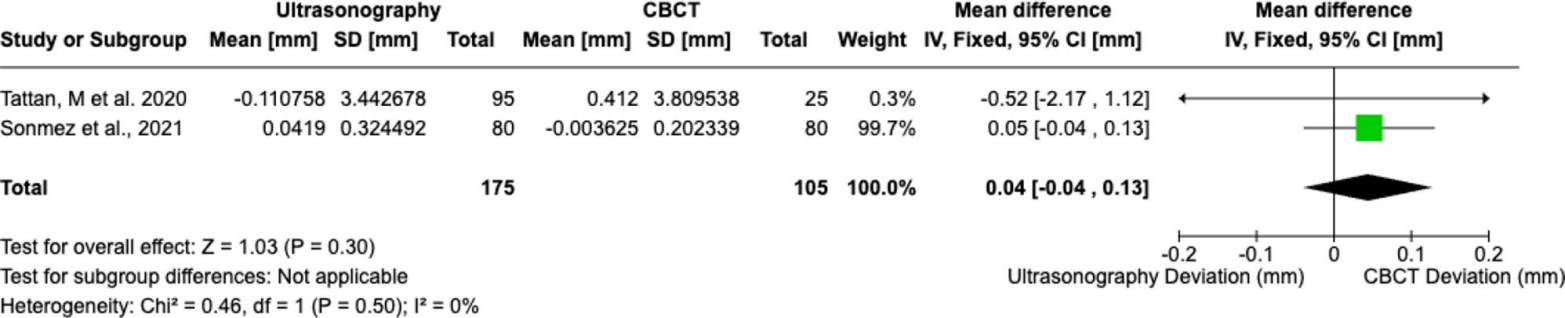
Soft tissue thickness detection

Schwinding et al., and Hilgenfield. et al. focused on the reproducibility of implant placement with three studies suitable for meta-analysis [16, 17]. MRI exhibited marginally greater deviations than CBCT in implant tip positioning (0.30 mm, 95% CI -0.08, 0.68), entry-level (0.38 mm, 95% CI, 0.04, 0.71), and angulation (0.81^°^, 95% CI, -0.50, 2.21) (Fig. 3a-c). For soft-tissue assessment, ultrasonography demonstrated superior accuracy to CBCT (mean difference: 0.04 mm, 95% CI, -0.04, 0.13) (Fig. 4) [21, 22].

### Narrative review of heterogenous results

Due to significant methodological heterogeneity, quantitative analysis was not statistically feasible for all included papers. Rodriguez Betancourt et al. compared the difference between guided ultrasonography and non-guided ultrasonography rather than on diagnostic accuracy with CBCT as a standard reference [18]. This team demonstrated that guided US scans could improve the accuracy of mapping hard and soft tissue, suggesting that this technique could potentially enhance soft tissue imaging quality compared to CBCT. Flick et al. demonstrated that 3T MRI can detect changes in sinus volume following augmentation, being excluded as no direct CBCT comparison was provided [19]. This group discovered the feasibility of MRI imaging technique as the radiation-free alternative with comparable accuracy to CBCT. Kang et al. used a zero-echo time MR system (MR Discovery 750 W, GE Healthcare) to visualize the inferior alveolar nerve with measurements matching those from CBCT [23, 25–27]. Sinjab et al. reported a mean correlation of bone ridge width 0.97 [20]; since it was the only study on crestal bone width, which gave determined accuracy of bone thickness in 3-dimension. Probst et al. reported mean deviations between virtually planned and resulting implant position (entry point 0.80 ± 0.30 mm, tip position 1.20 ± 0.60 mm, angular deviation 4.90 ± 3.60 degree), as well as occlusal surface deviations (0.25 ± 0.03 mm) using 3T MRI [28]. Whilst this provided similar outcomes to the literature included in the meta-analysis (REF) however was not included due to a lack of CBCT comparison. Hilgenfeld et al. observed higher root-mean-square (RMS) deviations in dental MRI (0.26 ± 0.10 mm) versus CBCT (0.10 ± 0.04 mm), though both remained clinically acceptable [24]. Since it was the only study on crestal bone width, it was excluded from the meta-analysis. Siqueira et al. highlighted US’s ability to evaluate peri-implant facial crestal bone dimension with 0.17 ± 0.23 mm deviation between Ultrasonography and CBCT [29]. Tattan et al. highlighted the mean difference between Ultrasonography and CBCT range from − 0.21 to 0.46 mm without statistical significance [21].

## Discussion

This review proposed a clinically relevant question, well-grounded in the clinical imperative to reduce patient radiation. In limiting studies to those published in English, involving human or in-vivo models, and dating from the past 10 years this review ensured relevance to contemporary clinical practice; but it may have introduced selection bias, potentially overlooking valuable data from diverse populations. Decisions to exclude studies with fewer than eight cases, while aimed at maintaining methodological rigor, may have omitted innovative pilot studies, limiting the scope of emerging technologies or niche applications. The central challenge was the substantial heterogeneity across included studies, stemming from inconsistent imaging protocols (e.g. MRI coil configurations, ultrasonographic frequencies), variable reference standards (e.g., surgical outcomes vs. soft-tissue thickness). These discrepancies hindered wider statistical analysis, necessitating the exclusion of multiple studies from the meta-analysis and the requirement for a narrative presentation of these heterogeneous results.

It should be noted that a number of the non-ionizing techniques provided Probst et al. used a 3T MRI system (Edition, Philips Healthcare) with a 16-channel Head and Neck spine array without the dedicated surface coils used in previous studies [15–17] making the results more applicable to routine practice. Standard protocol has not yet been established, though Hilgenfeld et al. demonstrated that optimising MRI sequences reduced geometric errors by 40% in edentulous models [30]. A previous meta-analysis reported an average error for CBCT-guided implant placement of 1.30 mm for implant tip positioning (95% CI: 0.98, 1.50), 1.00 mm for the entry level (95% CI: 0.75, 1.22), and 3.80° for angulation (95% CI: 3.18, 4.43) [31]. In our study, the pooled deviations were within acceptable error ranges for all parameters, with values of 0.30 mm (95% CI: − 0.08, 0.68) for implant tip placement, 0.38 mm (95% CI: 0.04, 0.71) for the entry level, and 0.81° (95% CI: − 0.50, 2.12) for implant angulation. These findings suggest that MRI treatment planning is a feasible and reliable alternative for dental implant treatment planning [11]. Moreover, to our knowledge, there is no previously pooled data evaluating the accuracy of MRI-guided implant placement.

Ultrasonography excels in soft-tissue evaluation, offering real-time, non-ionizing imaging critical for aesthetic planning and monitoring peri-implant pathologies. Gingival thickness is critical for implant and periodontal surgeries that require a high aesthetic outcome. According to Fig. 4, ultrasonography showed less deviation, but the difference was not statistically significant. Tattan et al. conducted a detailed analysis across six parameters: interdental papilla height, soft tissue height at teeth, mucosal thickness at teeth, soft tissue height at the edentulous ridge, mucosal thickness at the edentulous ridge, and crestal bone level [21]. The inter-rater correlation coefficients (ICC) for soft tissue height demonstrated good to excellent agreement (ranging from 0.654 to 0.918), confirming the reliable accuracy of ultrasonography [21]. When compared to direct measurements, Ultrasonography showed minimal differences (-0.015 mm to 0.479 mm) and strong agreement (ICC values), highlighting its precision and reliability in capturing critical periodontal measurements [21]. Ultrasonography offers a cost-effective and more accessible alternative to MRI, making it well-suited for clinical applications requiring detailed soft tissue evaluation. These applications could reduce reliance on CBCT in specific scenarios, such as serial post-operative evaluations or high-risk patients, or for the long-term review of palliative peri-implantitis cases both reducing dose metrics to the patient and the limitations of scatter and soft tissue accuracy from CBCT.

However, widespread adoption requires standardised imaging protocols and validation against surgical outcomes, which are currently lacking in the literature. RCTs comparing MRI- and CBCT-guided surgeries are critical. Consensus on MRI optimisation (artefact reduction, scan time) and ultrasonographic penetration enhancement is needed. Clinicians should advocate for interdisciplinary collaboration to refine these technologies.

Limitations.

Only 12 studies met the inclusion criteria, and most had small sample sizes (median = 18 participants), which limits statistical power and generalisability. 50% of included studies did not report whether patients were recruited consecutively or randomly, introducing a risk of selection bias. Third, heterogeneity was substantial (I² = 72% for nerve-canal distance; 68% for ridge width), largely attributable to variability in MRI sequences (T1-w vs. STIR) and US probe frequencies (5–12 MHz). Finally, the meta-analysis employed study-level, not individual-level, data, precluding adjustment for confounders such as bone density or implant site.

## Conclusion

Within the limitations of the available evidence, ultrasonography (US) and MRI demonstrated clinically acceptable accuracy for specific measurements (e.g. alveolar ridge width, nerve canal position). However, the current evidence base is small and heterogeneous; therefore, these modalities should be considered promising alternatives rather than full replacements for CBCT in routine implant planning. Further multicentre trials using standardised imaging protocols are required.

## Electronic supplementary material

Below is the link to the electronic supplementary material.

Supplementary Material 1

## Data Availability

No datasets were generated or analysed during the current study.
