# Supplementary material for: Are dental magnetic resonance imaging and ultrasonography techniques reliable alternatives for treatment planning dental implants? A systematic review and meta-analysis
Source: Int J Implant Dent. 2025 Aug 11;11:52. doi: 10.1186/s40729-025-00634-6 (PMC12339830; doi:10.1186/s40729-025-00634-6)
Supplement: Supplementary file 1 — Supplementary Material 1 [file 40729_2025_634_MOESM1_ESM.docx]

**Supplementary data：**

Ovid MEDLINE(R) Epub Ahead of Print and In-Process, In-Data-Review &amp; Other Non-Indexed

Citations and Daily &lt;August 20, 2024&gt;

1 exp Magnetic Resonance Imaging/ or Diffusion Tensor Imaging/ or (MRI or Magnetic

resonance imag* or MR imag* or MR scan or NMR or maxillofacial imaging or ultrasonography

or neurography or visuali?ation or non-ionising imaging or non-radiative imaging).mp.

2 exp Dental Implantation/

3 exp Dental Implants/ or exp Mandibular Nerve/ or Trigeminal Nerve/ or (sinus lift or sinus

lifting or permanent dental restoration).mp. or (dent* and (implant* or art?fact* or bone

augmentation or bone graft or bone reconstruction)).mp.

4 exp dental materials/ or dental material*.mp.

5 2 or 3 or 4

6 1 and 5

7 exp Machine Learning/ or (deep learning or machine learning).mp.

8 exp Artificial Intelligence/ or (artificial intelligence or AI or computational or computer-

assisted or algorithm* or CNN or convolutional or neural network*).mp.

9 (art?fact* adj2 (reduc* or size or correct*)).mp.

10 Image Enhancement/ or Signal-To-Noise Ratio/ or (image quality or image process* or

clarity or accuracy or feasibility).mp.

11 7 or 8 or 9 or 10

12 6 and 11

13 limit 12 to (yr=&quot;2014 -Current&quot; and (chinese or english))

BIOSIS Citation Index (via Web of Science)

((&quot;Magnetic Resonance Imaging&quot; OR &quot;Diffusion Tensor Imaging&quot; OR (MRI OR &quot;Magnetic

resonance imag*&quot; OR &quot;MR imag*&quot; OR &quot;MR scan&quot; OR NMR OR &quot;maxillofacial imaging&quot; OR

ultrasonography OR neurography OR visuali$ation OR &quot;non-ionising imaging&quot; OR &quot;non-radiative

imaging&quot;) ) AND ((&quot;Dental Implantation&quot; ) OR (&quot;Dental Implants&quot; OR &quot;Mandibular Nerve&quot; OR

&quot;Trigeminal Nerve&quot; OR (&quot;sinus lift&quot; OR &quot;sinus lifting&quot; OR &quot;permanent dental restoration&quot;) OR

(dent* AND (implant* OR art$fact* OR &quot;bone augmentation&quot; OR &quot;bone graft&quot; OR &quot;bone

reconstruction&quot;)) ) OR (&quot;dental materials&quot; OR &quot;dental material*&quot;) ) ) AND ((&quot;Machine Learning&quot;

OR (&quot;deep learning&quot; OR &quot;machine learning&quot;) ) OR (&quot;Artificial Intelligence&quot; OR (&quot;artificial

intelligence&quot; OR AI OR computational OR computer-assisted OR algorithm* OR CNN OR

convolutional OR &quot;neural network*&quot;) ) OR ((art$fact* NEAR/2 (reduc* OR size OR correct*)) )

OR (&quot;Image Enhancement&quot; OR &quot;Signal-To-Noise Ratio&quot; OR (&quot;image quality&quot; OR &quot;image

process*&quot; OR clarity OR accuracy OR feasibility) ) ) (Topic) and 2024 or 2023 or 2022 or 2021

or 2020 or 2019 or 2018 or 2017 or 2016 or 2015 or 2014 (Publication Years)

Scopus

( TITLE ( ( ( &quot;Magnetic Resonance Imaging&quot; OR &quot;Diffusion Tensor Imaging&quot; OR ( mri OR

&quot;Magnetic resonance imag*&quot; OR &quot;MR imag*&quot; OR &quot;MR scan&quot; OR nmr OR &quot;maxillofacial imaging&quot;

OR ultrasonography OR neurography OR visuali*ation OR &quot;non-ionising imaging&quot; OR &quot;non-

radiative imaging&quot; ) ) AND ( ( &quot;Dental Implantation&quot; ) OR ( &quot;Dental Implants&quot; OR &quot;Mandibular

Nerve&quot; OR &quot;Trigeminal Nerve&quot; OR ( &quot;sinus lift&quot; OR &quot;sinus lifting&quot; OR &quot;permanent dental

restoration&quot; ) OR ( dent* AND ( implant* OR art*fact* OR &quot;bone augmentation&quot; OR &quot;bone graft&quot;

OR &quot;bone reconstruction&quot; ) ) ) OR ( &quot;dental materials&quot; OR &quot;dental material*&quot; ) ) ) AND ( (

&quot;Machine Learning&quot; OR ( &quot;deep learning&quot; OR &quot;machine learning&quot; ) ) OR ( &quot;Artificial Intelligence&quot;

OR ( &quot;artificial intelligence&quot; OR ai OR computational OR computer-assisted OR algorithm* OR

cnn OR convolutional OR &quot;neural network*&quot; ) ) OR ( ( art*fact* W/2 ( reduc* OR size OR correct*

) ) ) OR ( &quot;Image Enhancement&quot; OR &quot;Signal-To-Noise Ratio&quot; OR ( &quot;image quality&quot; OR &quot;image

process*&quot; OR clarity OR accuracy OR feasibility ) ) ) ) OR ABS ( ( ( &quot;Magnetic Resonance

Imaging&quot; OR &quot;Diffusion Tensor Imaging&quot; OR ( mri OR &quot;Magnetic resonance imag*&quot; OR &quot;MR

imag*&quot; OR &quot;MR scan&quot; OR nmr OR &quot;maxillofacial imaging&quot; OR ultrasonography OR neurography

OR visuali*ation OR &quot;non-ionising imaging&quot; OR &quot;non-radiative imaging&quot; ) ) AND ( ( &quot;Dental

Implantation&quot; ) OR ( &quot;Dental Implants&quot; OR &quot;Mandibular Nerve&quot; OR &quot;Trigeminal Nerve&quot; OR (

&quot;sinus lift&quot; OR &quot;sinus lifting&quot; OR &quot;permanent dental restoration&quot; ) OR ( dent* AND ( implant* OR

art*fact* OR &quot;bone augmentation&quot; OR &quot;bone graft&quot; OR &quot;bone reconstruction&quot; ) ) ) OR ( &quot;dental

materials&quot; OR &quot;dental material*&quot; ) ) ) AND ( ( &quot;Machine Learning&quot; OR ( &quot;deep learning&quot; OR

&quot;machine learning&quot; ) ) OR ( &quot;Artificial Intelligence&quot; OR ( &quot;artificial intelligence&quot; OR ai OR

computational OR computer-assisted OR algorithm* OR cnn OR convolutional OR &quot;neural

network*&quot; ) ) OR ( ( art*fact* W/2 ( reduc* OR size OR correct* ) ) ) OR ( &quot;Image Enhancement&quot;

OR &quot;Signal-To-Noise Ratio&quot; OR ( &quot;image quality&quot; OR &quot;image process*&quot; OR clarity OR accuracy

OR feasibility ) ) ) ) ) AND PUBYEAR &gt; 2013 AND PUBYEAR &lt; 2025 AND ( LIMIT-TO (

LANGUAGE , &quot;English&quot; ) OR LIMIT-TO ( LANGUAGE , &quot;Chinese&quot; ) )

Cochrane Library - Cochrane Central Register of Controlled Trials (CENTRAL)

(([mh &quot;Magnetic Resonance Imaging&quot;] OR [mh ^&quot;Diffusion Tensor Imaging&quot;] OR (MRI:ti,ab,kw

OR (&quot;Magnetic resonance&quot; NEXT imag*):ti,ab,kw OR (&quot;MR&quot; NEXT imag*):ti,ab,kw OR &quot;MR

scan&quot;:ti,ab,kw OR NMR:ti,ab,kw OR &quot;maxillofacial imaging&quot;:ti,ab,kw OR

ultrasonography:ti,ab,kw OR neurography:ti,ab,kw OR visuali?ation:ti,ab,kw OR &quot;non-ionising

imaging&quot;:ti,ab,kw OR &quot;non-radiative imaging&quot;:ti,ab,kw) ) AND (([mh &quot;Dental Implantation&quot;] ) OR

([mh &quot;Dental Implants&quot;] OR [mh &quot;Mandibular Nerve&quot;] OR [mh ^&quot;Trigeminal Nerve&quot;] OR (&quot;sinus

lift&quot;:ti,ab,kw OR &quot;sinus lifting&quot;:ti,ab,kw OR &quot;permanent dental restoration&quot;:ti,ab,kw) OR

(dent*:ti,ab,kw AND (implant*:ti,ab,kw OR art?fact*:ti,ab,kw OR &quot;bone augmentation&quot;:ti,ab,kw

OR &quot;bone graft&quot;:ti,ab,kw OR &quot;bone reconstruction&quot;:ti,ab,kw)) ) OR ([mh &quot;dental materials&quot;] OR

(&quot;dental&quot; NEXT material*):ti,ab,kw) ) ) AND (([mh &quot;Machine Learning&quot;] OR (&quot;deep

learning&quot;:ti,ab,kw OR &quot;machine learning&quot;:ti,ab,kw) ) OR ([mh &quot;Artificial Intelligence&quot;] OR

(&quot;artificial intelligence&quot;:ti,ab,kw OR AI:ti,ab,kw OR computational:ti,ab,kw OR computer-

assisted:ti,ab,kw OR algorithm*:ti,ab,kw OR CNN:ti,ab,kw OR convolutional:ti,ab,kw OR

(&quot;neural&quot; NEXT network*):ti,ab,kw) ) OR ((art?fact*:ti,ab,kw NEAR/2 (reduc*:ti,ab,kw OR

size:ti,ab,kw OR correct*:ti,ab,kw)) ) OR ([mh ^&quot;Image Enhancement&quot;] OR [mh ^&quot;Signal-To-

Noise Ratio&quot;] OR (&quot;image quality&quot;:ti,ab,kw OR (&quot;image&quot; NEXT process*):ti,ab,kw OR

clarity:ti,ab,kw OR accuracy:ti,ab,kw OR feasibility:ti,ab,kw) ) )

Limit to 2024-2024
